# Hydrophobicity causes anomalous migration of cystine/glutamate antiporter SLC7A11 in SDS‐PAGE with low acrylamide concentration

**DOI:** 10.1002/2211-5463.70019

**Published:** 2025-03-24

**Authors:** Nsengiyumva Emmanuel, Qian He, Yixin Kang, Dianbao Zhang, Min Gao, Minglin Wang, Kexin Fan, Jingwen Xiong, Shaobo Wu, Botao Fa, Zhengtao Xiao, Yingfang Niu, Jun Yao, Yilei Zhang

**Affiliations:** ^1^ Department of Biochemistry and Molecular Biology, School of Basic Medical Sciences Xi'an Jiaotong University China; ^2^ Department of Biomedical Laboratory Sciences, School of Health Sciences, College of Medicine and Health Sciences University of Rwanda Kigali Rwanda; ^3^ Henan Key Laboratory of Cancer Epigenetics, Cancer Institute, The First Affiliated Hospital College of Clinical Medicine of Henan University of Science and Technology Luoyang China; ^4^ School of Management/Guangdong‐Hong Kong‐Macao Greater Bay Area Cultural and Tourism Integration Innovation Development Research Center Shenzhen Polytechnic University China

**Keywords:** acrylamide gel concentration, gel mobility, hydrophobicity, SDS‐PAGE, SLC7A11, transmembrane domain

## Abstract

The cystine/glutamate antiporter, solute carrier family 7 member 11 (SLC7A11), plays a crucial role in regulating redox homeostasis and cell death processes such as apoptosis and ferroptosis. These processes are implicated in various diseases, including cancer, organ injuries and neurodegenerative disorders. However, the sodium dodecyl sulfate‐polyacrylamide gel electrophoresis (SDS‐PAGE) expression pattern of SLC7A11 varies across studies and remains unclear. In many studies, including ours, SLC7A11 migrates at an atypical molecular weight (MW) of approximately 37 kDa, which is lower than its theoretical molecular mass of 55.4 kDa. This discrepancy raises concerns about the precise molecular mass and expression pattern of SLC7A11 in SDS‐PAGE. We confirmed that this fast‐migrating band corresponds to SLC7A11 through knockdown of endogenous SLC7A11 or overexpression of exogenous SLC7A11. Furthermore, we ruled out the possibility of proteolytic cleavage after protein translation. We found that the high hydrophobicity of SLC7A11 is a key factor responsible for its anomalous migration. Substituting the non‐polar residue isoleucine (Ile) with the polar residue asparagine (Asn) reduced its hydrophobicity and restored normal migration, aligning with its predicted MW of 55 kDa. Additionally, we observed that SLC7A11 migrated faster in SDS‐PAGE at lower acrylamide concentrations, whereas higher concentrations (e.g. 12% or 15%) eliminated the gel shift. This study clarifies the expression pattern of SLC7A11 in SDS‐PAGE and emphasizes the importance of considering physicochemical properties such as hydrophobicity and gel concentration when characterizing membrane proteins like SLC7A11.

## Graphical abstract

SLC7A11 frequently migrates faster in SDS‐PAGE. The present study found that the high hydrophobicity of SLC7A11 causes its anomalous migration in SDS‐PAGE with a low concentration of acrylamide gel. Replacing isoleucine with asparagine reduced hydrophobicity and restored its normal migration at 55 kDa, revealing the role of hydrophobicity and gel concentration in gel shift of SLC7A11.

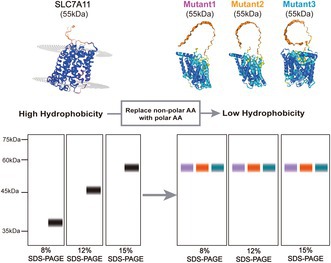

**Abbreviations**
CDS: coding sequence
CCDS: Consensus Coding Sequence
DMEM: Dulbecco's modified Eagle's medium
FMD: fractionation mass deviation
NCBI: National Center for Biotechnology Information
PTM: post‐translational modification
PVDF: poly(vinylidene difluoride)
SLC3A2: solute carrier family 3 member 2
SLC7A11: solute carrier family 7 member 11
TM: transmembrane
TMD: transmembrane domain
WB: western blot

Solute carrier family 7 member 11 (SLC7A11), also known as xCT, is the light chain subunit of the heterodimeric cystine/glutamate antiporter system Xc‐, in combination with the heavy chain subunit, 4F2hc/solute carrier family 3 member 2 (SLC3A2). The system Xc‐ plays a crucial role in cellular redox homeostasis by mediating the exchange of extracellular cystine for intracellular glutamate in a 1 : 1 ratio. The imported cystine is then reduced to cysteine, which is essential for the synthesis of glutathione, a major cellular antioxidant that protects cells from oxidative stress‐induced cell death, such as apoptosis and ferroptosis [1, 2, 3, 4]. Dysregulation of SLC7A11 has been linked to various pathological conditions, including cancer, tissue injury and neurodegerative diseases. The dysregulation of SLC7A11 has been associated with tumor development upon inactivation of tumor suppressors such as p53, BAP1 and KEAP1 [5, 6, 7]. SLC7A11 has also been shown to play a role in regulating inflammation and promoting tissue repair. Dysregulation of SLC7A11 can impair wound healing and contribute to chronic inflammation, which in turn plays a role in the progression of various diseases. For example, studies have shown that decreased expression of SLC7A11 is associated with liver injury, kidney damage and lung injury in various disease states [8, 9]. Moreover, dysfunctional SLC7A11 has been linked to the pathogenesis of neurodegenerative diseases, such as Alzheimer's and Parkinson's disease, which are characterized by impaired glutathione metabolism and lipid peroxidation [10]. Therefore, the accurate determination of SLC7A11 protein levels and its molecular characterization are crucial for elucidating its physiological function, regulatory mechanisms and potential therapeutic interventions.

SDS‐PAGE coupled with western blot (WB) analysis is a widely used proteomics technique for protein separation, molecular mass estimation and analysis. Although most proteins migrate to the molecular mass position corresponding to their molecular size on SDS‐PAGE, some proteins deviate from their expected molecular mass, either migrating faster or slower than their calculated molecular mass. This phenomenon is referred to as a ‘gel shift’ [11, 12, 13, 14]. Protein gel mobility shifts can be influenced by various factors, including the expression of different protein isoforms, post‐translational modifications (PTMs) (e.g. glycosylation, phosphorylation, acetylation, lipidation), proteolytic cleavage, physicochemical properties, detergent binding capacity and detergent–protein interactions [13, 15, 16]. For example, water‐soluble proteins typically migrate to their correct molecular mass positions on SDS‐PAGE [17], whereas integral transmembrane (TM) proteins, which make up 20–30% of human open reading frames [18, 19], often exhibit anomalous migration [13, 20]. This deviation from the actual molecular size for integral membrane proteins is primarily attributed to their physicochemical properties, particularly high hydrophobicity, SDS detergent binding capacity and detergent‐protein conformational changes. Additionally, the polyacrylamide gel concentration can influence the migration of membrane proteins in SDS‐PAGE, probably as a result of the helical structures within these proteins [12, 21]. The anomalous electrophoretic mobility complicates the accurate determination of a protein's molecular size and, when observed, should be investigated with caution. Previous studies, including ours, have reported that the SLC7A11 protein migrates faster in SDS‐PAGE, with an apparent molecular mass of approximately 37 kDa, which is lower than its theoretical molecular mass of 55.4 kDa [5, 22, 23, 24]. However, various antibodies against SLC7A11 suggest a molecular mass of 55 kDa, probably as a result of non‐specific binding of the antibodies [25]. Therefore, it is crucial to resolve the precise molecular mass of SLC7A11 and clarify its expression pattern in SDS‐PAGE.

The present study unraveled the mystery of the apparent molecular mass deviation of SLC7A11 on SDS‐PAGE and the underlying electrophoretic gel shift mechanisms. The high hydrophobicity of SLC7A11, evidenced by high GRAVY score index and high composition non‐polar amino acid residues, was demonstrated as the reason for its atypical low molecular mass display on SDS‐PAGE. Substituting the hydrophobic residue, isoleucine (Ile) with polar residue asparagine (Asn) in either TM domain (TMD) or extramembrane region, significantly restore its normal migration on SDS‐PAGE, aligning with its calculated molecular mass of approximately 55.4 kDa. Furthermore, increasing the acrylamide gel concentration significantly decreased the migration deviation of SLC7A11. Overall, our findings elucidate the mechanisms underlying the migration of SLC7A11 in SDS‐PAGE.

## Materials and methods

### Data source and bioinformatics analysis

The genomic data for SLC7A11 were retrieved from the National Center for Biotechnology Information (NCBI) database (https://www.ncbi.nlm.nih.gov; accessed 26 March 2024). The SLC7A11 CCDS report and coding sequence (CDS) were retrieved from the Consensus Coding Sequence (CCDS) database (https://www.ncbi.nlm.nih.gov/CCDS/CcdsBrowse.cgi). The mass spectrometry peptide data and sequence coverage were sourced from the Human Peptide Atlas database (https://peptideatlas.org). The virtual 1‐SDS‐PAGE/WB migration patterns were acquired from the PUMBA database [20], a repertoire for accurate human protein gel migration patterns (https://pumba.dcsr.unil.ch). The proteolytic cleavage site prediction of SLC7A11 was conducted using the SitePrediction webserver [26] (https://www.dmbr.ugent.be/prx/bioit2‐public/SITEPREDICTION), with human protease enzymes from the MEROPS database (https://www.ebi.ac.uk/merops/index.shtml). The physicochemical properties of the SLC7A11 protein were determined using the ProtParam tool (https://web.expasy.org/protparam). The TMD sequences were accessed from the Human Transmembrane Proteome database (https://htp.unitmp.org). UMRC6 cells were collected in triplicates and subjected for Third‐generation sequencing by the BioMarker Technologies Company (Beijing, China).

### Plasmid DNA constructs

To generate the pLVX‐Flag‐SLC7A11, the human *SLC7A11* coding DNA sequence was amplified and tagged with Flag tag (DYKDDDDK) at N terminals by PCR amplification using a plasmid containing *SLC7A11* cDNA as a template. Amplification was performed with the forward primer containing *Eco*RI restriction cutting site and Flag tag sequence (5′‐cggaattccgATGGATTACAAGGACGACGATGACAAGATGGTCAGAAAGCCTGTTGTGTCC‐3′) and the reverse primer containing the *Xba*I restriction cutting site (5′‐gcatctagaTCATAACTTATCTTCTTCTGGTACAACTTCCAGTATTATTTGTAATG‐3′). The PCR program included: initial denaturation at 98 °C for 45 s, 30 cycles of denaturation at 98 °C for 10 s, annealing at 58 for 30 s and elongation at 72 °C for 20 s, a final elongation at 72 for 5 min, and a hold at 4 °C. The amplified Flag‐SLC7A11 DNA sequence was cloned into *Eco*RI and *Xba*I sites of lentivirus expression vector pLVX‐Puro. Both 1 μg of insert DNA and the vector DNA were cut with QuickCut *Eco*RI (Takara, Shiga, Japan) and QuickCut *Xba*I (Takara) restriction cutting enzymes, separated on 1% agarose gel and purified using the GeneJET™ Gel extraction kit (Thermo Fisher Scientific, Waltham, MA, USA). Then, the cut DNAs were ligated by T7 DNA ligase (NEB, Ipswich, MA, USA) with a 3 : 1 (insert: vector) ligation ratio in T7 DNA ligase buffer (NEB) for 30 min at 25 °C. The 5 μL of ligation reactions were transformed into 50 μL of competent DH5α bacteria cells. The transformed bacteria were grown overnight (14–16 h) at 37 °C. Plasmid DNAs were extracted from transformed bacteria using the TIANprep Mini Plasmid Kit (Tiangen, Beijing, China) and all constructs were validated by DNA sanger sequencing. Plvx‐SLC7A11‐Myc‐Flag and *SLC7A11* shRNAs containing expression vectors have been described previously [27]. The SLC7A11 hydrophobic mutant plasmid DNAs (in PLVX‐puro) were synthesized by Tsingke Biotech Co., Ltd (Beijing, China); the mutant plasmids were tagged with Myc and Flag tags at the C‐terminal. Mutant I (SLC7A11^Mut1^, XA0149031‐1), includes 12 mutations in TMDs (one in each TMD, I52N, I82N, I133N, I168N, I205N, I232N, I276N, I321N, I370N, I400N, I427N and I456N), Mutant II (SLC7A11^Mut2^, XA0149031‐2), contains 12 mutations in extramembrane regions (I10N, I104N, I114N, I150N, I190N, I214N, I266N, I292N, I408N, I479N, I484N and I490N) and Mutation III (SLC7A11^Mut3^, XA0149031‐3) combines mutations of both Mutant I and Mutation II. Mutants were designed by ensuring that the predicted molecular mass [~55.4 kDa] and physicochemical properties other than hydrophobicity are the same among all mutants and the wild‐type. All constructs were validated by Sanger sequencing.

### Cell culture and plasmid transfection

The 293T (RRID: CVCL_0063) and UMRC6 (RRID: CVCL_2741) cells were cultured in Dulbecco's modified Eagle's medium (DMEM) (Thermo Fisher Scientific) supplemented with 10% fetal bovine serum, 10 000 U·L^−1^ penicillin–streptomycin at 37 °C in a humidified incubator containing 5% of CO_2_ gas. Plasmids were transiently transfected using poly(ethylenimine) (Yeasen, Shanghai, China) transfection reagent. Briefly, 9 μg of poly(ethylenimine) was diluted in 150 μL of Opti‐MEM reagent (Gibco, Waltham, MA, USA) and 3 μg of plasmid was diluted in 150 μL of Opti‐MEM reagent. We added the poly(ethylenimine) to diluted plasmid DNAs and incubated at room temperature for 30 min. The transfection mixtures were then added to 293T cells grown to 70–90% confluency in new DMEM media and then incubated at 37 °C in a 5% CO_2_ incubator. After 8 h of transfection, the culture media was changed to new DMEM media and returned at 37 °C to a 5% CO_2_ incubator for 48 h.

### Protein extraction and concentration quantification

The grown 293T cells were washed with 1 mL of cold phosphate‐buffered saline three times and lysed in NP‐40 lysis buffer (50 mm Tris, pH 7.4, 250 mm NaCl, 5 mm EDTA, 50 mm NaF, 1 mm Na_3_VO_4_ and 1% Nonidet P40) containing complete mini protease inhibitors (C0001; TargetMol, Boston, MA, USA), phosphatase inhibitor cocktail I (C0002; TargetMol) and phosphatase inhibitor cocktail II (C0003; TargetMol). The cells were sonicated on ice with an ultrasonic homogenizer set at 15% power, ON for 5 s, OFF for 5 s, and repeated three times. Then, the cell lysates were centrifuged at 13680 g for 15 min at 4 °C. The upper fluids were transferred to a clean microcentrifuge tube and stored in a freezer at −20 °C. The protein concentration of cell lysates was quantified using a BCA Protein Assay Kit (Life Technologies, Carlsbad, CA, USA) and BSA standards were used to generate accurate standard curves for concentration calculations.

### SDS‐PAGE

Protein samples were separated on SDS‐PAGE. For this, 1 μg of total cell lysates was prepared in 1× laemmli loading buffer (1 m Tris‐HCl, pH 6.5, 1 m dithiothreitol, 2% SDS, 10% glycerol, 0.01% bromophenol blue). For non‐reducing SDS‐PAGE, dithiothreitol was omitted from the laemmli loading buffer. The cell lysates in the loading buffer were denatured at either 37 °C for 15 min or 95 °C for 5 min, and then spun down a using minicentrifuge. Then, 30 μL (30 μg) of denatured protein samples was loaded and separated in either 8% or 10% resolving gels [30% acrylamide‐bisacrylamide (29 : 1), 375 mm Tris HCl, pH 8.8, 0.1% SDS 0.1% ammonium persulfate and 0.067% *N*,*N*,*N*′,*N*′‐tetramethylethylenediamine]. For the SLC7A11 migration patterns analysis on acrylamide gel concentration, the protein samples were separated in 6%, 8%, 10%, 12% and 15% SDS‐PAGE. The stacking gel was 5% [30% acrylamide‐bisacrylamide (29 : 1), 125 mm Tris‐HCl, pH 6.8, 0.1% SDS, 0.1% ammonium persulfate and 0.125% *N*,*N*,*N*′,*N*′‐tetramethylethylenediamine]. The electrode buffer contained 25 mm Tris, 0.192 mm glycine and 0.1% SDS. The SDS‐PAGE was calibrated with prestained protein markers [PageRuler Prestained Protein Ladder 180 (Thermo Fisher Scientific); ColorMixed protein marker 180 kDa (ABclonal, Woburn, MA, USA); protein marker 10–250 kDa (Epizyme, Cambridge, MA, USA); StarRuler Color Prestained Protein Marker 10–180 kDa (GenStar, San Francisco, CA, USA)] and used unheated. The electrophoresis was run at 80 V and 400 mA for 30 min until the samples moved out of stacking gel, and then this was changed to 120 V for 120 min until the blue loading dye was about to move out of the gel. For the SDS concentration‐dependent migration patterns experiment, different amounts of SDS were added to the laemmli buffer: 2% (0.8 g), 1% (0.4 g), 0.5% (0.2 g), 0.25% (0.1 g) and 0% (no SDS) in 10 mL 4× buffer.

### Western blotting

Total SDS‐PAGE separated proteins in the gel were electrophoretically transferred to a poly(vinylidene difluoride) (PVDF) membrane (Immobilon®; Sigma‐Aldrich, St Louis, MO, USA) in a mini trans‐blot cell (Bio‐Rad, Hercules, CA, USA) at 400 mA in cold transfer buffer with alcohol for 2 h. After the transfer, the membranes were blocked with a 5% blotting grade‐blocker (Beyotime, Haimen, China) in TBS‐T (20 mm Tris‐base, 150 mm NaCl, pH 7.6, 0.1% Tween‐20) for 1 h, followed by overnight incubation with primary antibodies at 4 °C on a shaking platform. The primary antibodies used were mouse‐anti‐tubulin (dilution 1 : 1000; Proteintech, Roemont, IL, USA), rabbit‐anti‐vinculin (dilution 1 : 1000; Cell Signaling Technology, Danvers, MA, USA), rabbit‐anti‐SLC7A11 (dilution 1 : 1000; Cell Signaling Technology) anti‐FLAG (dilution 1 : 1000; Beyotime), rabbit‐anti‐SLC3A2 (1 : 1000; Cell Signaling Technology) and mouse‐anti‐Myc (dilution 1 : 1000; Cell Signaling Technology). After three washes of 10 min with TBS‐T at room temperature, the PVDF membrane was then incubated with horseradish peroxidase‐conjugated secondary antibodies (goat‐anti‐rabbit or goat‐anti‐mouse, 1 : 5000; Thermo Fisher Scientific) on a rotating plate for 60 min at ambient temperature. Next, the PVDF membranes were rewashed with TBS‐T three times for 10 min at ambient temperature and then the membranes were treated with WB ECL detection reagents (1 : 1 ratio for reagents A and B; Novonature, Xi'an, Shaanxi, China) and imaged using a chemiluminescence gel documentation system. For reprobing with additional primary antibodies, the membranes were stripped at ambient temperature for 1 h with the stripping solution (CWBIO, Taizhou, China) and washed with TBS‐T for 10 min three times. The probing procedures from the membrane blocking step were then repeated.

### Proteolytic cleavage inhibition

The transiently transfected 293T cells with pLVX‐SLC7A11‐Myc‐Flag plasmid DNA or an empty vector (pLVX‐puro) in DMEM at 37 °C, in a 5% CO_2_ incubator were treated with protease inhibitor drugs; serine and cysteine proteases inhibitor, leupeptin hemisulfate, 50 μm (T6564; TargetMol); metalloproteinases inhibitor, Ilomastat, 50 μm (T2743, TargetMol); caspases inhibitor, Z‐VAD‐FMK, 10 μm (S7023; Selleck, Houston, TX, USA); or autophagy inhibitor, chloroquine, 10 μm (T8689, TargetMol). DMSO (Sigma‐Aldrich) was used as a control. The treated 293 T cells were grown for 48 h. Then, the cell lysates were separated via 10% SDS‐PAGE and analyzed with WB using anti‐SLC7A11 antibodies.

### Measurement of relative mobility and mobility retardation

The migration distance of SLC7A11, tubulin (used as control) and the protein marker (corresponding to a molecular mass of around 55 kDa) were measured using a ruler from the top of the resolving gel to the dye front. The relative mobility (*R*_f_) was calculated by dividing the migration distance of a protein by the migration distance of the dye front. The logarithm of relative mobility (log*R*_f_) values obtained for different SDS‐PAGE concentrations were plotted against gel concentration (*T*) to generate a Ferguson plot (matching the formula below). We calculated the mobility retardation (Δ*R*_f_) by subtracting the relative mobility of the reference protein (protein marker of ~55 kDa) from the relative mobility of the analyte protein (SLC7A11, tubulin). To ensure reproducibility, at least three independent SDS‐PAGE runs were conducted for each gel concentration.

$$\log R_{f}=K_{r}T+\log Y_{0}$$

*Y*_0_ represents the relative mobility at zero gel concentration and *K*_r_ is the coefficient of retardation.

## Results

### Anomalous migration of SLC7A11 in SDS‐PAGE

**Fig. 1:**
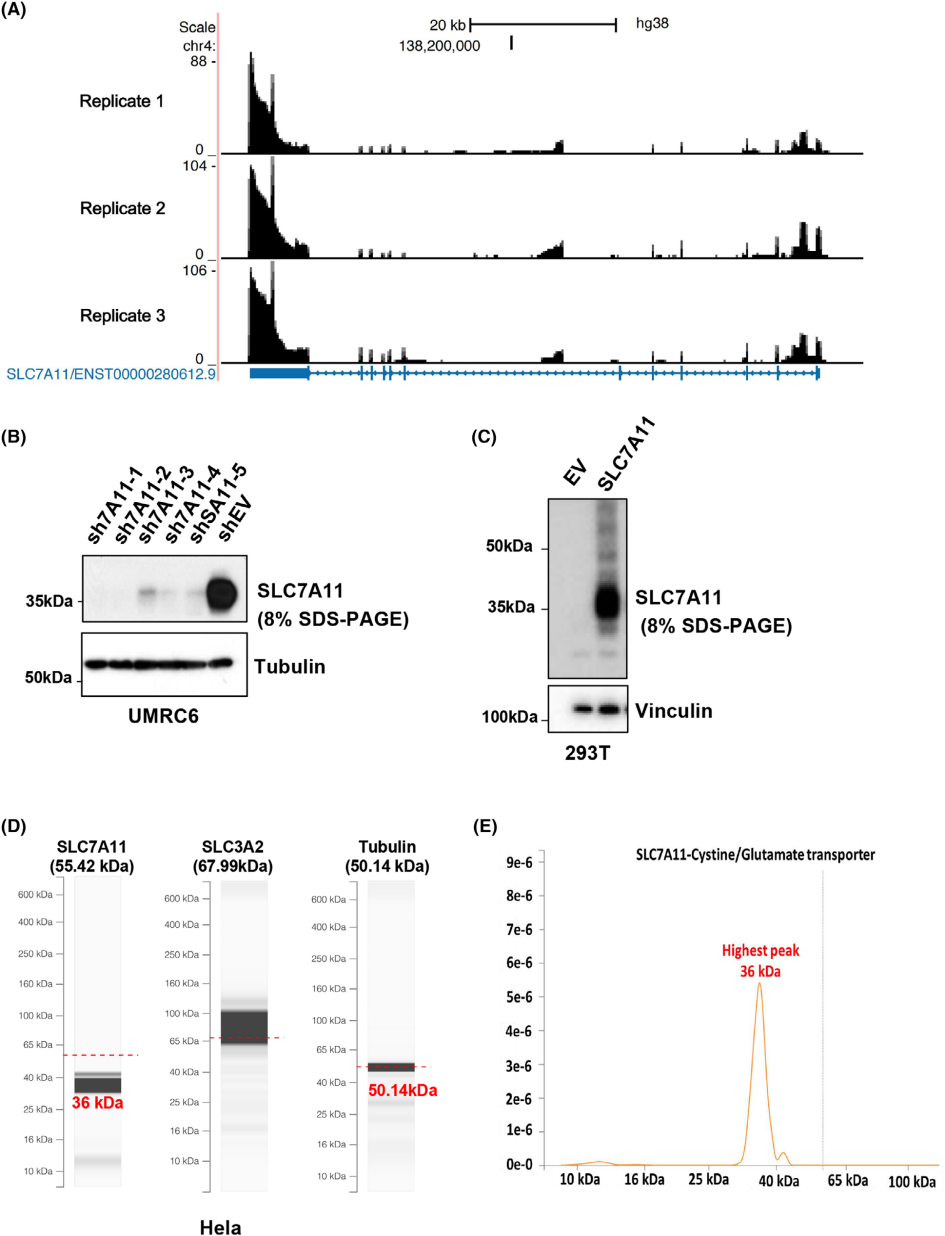
Solute carrier family 7 member 11 (SLC7A11) undergoes gel shift on SDS‐PAGE. (A) Third‐generation RNA sequencing for SLC7A11 reveals a single SLC7A11 transcript encompassing the complete sequence detected in the UMRC6 cell line. (B) WB for endogenous SLC7A11 (SLC7A11 knockdown was performed as a control for endogenous SLC7A11 expression). The experiment was repeated at least three times independently, whereas representative data are shown in the text. (C) WB for exogenous SLC7A11 (recombinant SLC7A11, SLC7A11‐Myc‐Flag). The experiment was repeated at least three times independently, whereas representative data are shown in the text. (D) PUMBA database‐generated SDS‐PAGE virtual WB migration patterns for SLC7A11 [Solute carrier family 3 member 2 (SLC3A2) and tubulin were used as a control]. (E) The SLC7A11 predominant migration pattern shows the highest peak of 36 kDa, which is the molecular mass of the primary, quantitatively dominant peptides bands in conventional gel or WB detection.

The human *SLC7A11* gene (NC_000004.12) is at chromosome 4q28.3, spanning from 138 164 097 to 138 312 671 base pairs and encompassing a DNA sequence of 148 575 bp. Alternative splicing enables the transcription of multiple unique mRNA transcripts from a single gene, resulting in various protein isoforms of distinct sizes [28, 29]. This possibility prompted us to conduct a comprehensive genomic analysis of SLC7A11 aiming to identify the predominant transcript in humans. First, we analyzed curated human *SLC7A11* genomic data from various databases. In addition to the annotated full‐length canonical transcript of SLC7A11 (NM_014331.1), which spans approximately 9645 bp, with an ORF of 12 exons consisting of 1506 nucleotides (CCDS3742.1), encoding the SLC7A11 protein of 501 amino acids (NCBI: NP_055146.1, UniProt: Q9UPY5), the NCBI database predicts seven alternative *SLC7A11* transcript variants potentially encoding different protein isoforms with different molecular sizes (Fig. S1A). Comparative analysis of these transcript variants to canonical transcript indicated that the exons 1 to 11 are conserved across the predicted transcripts X1 and 2, 3, 4 and 7. By contrast, the shortest transcript variants X5 and X6 solely encompass exons 1 to 6 encoding shorter SLC7A11 protein variants (34.7 and 31.1 kDa respectively) (Fig. S1A). Furthermore, we determined the SLC7A11 annotated coding sequence in the CCDS database; despite the annotated different predicted SLC7A11 transcript variants, the CCDS report for SLC7A11 indicates a single coding sequence, CCDS3742.1, which has been reported throughout different CCDS releases (Fig. S1B). We conducted third‐generation RNA sequencing in the UMRC6 cell line with three independent replicates, revealing transcription across all exons of SLC7A11 with rare instances of alternative splicing forms (Fig. 1A).

To characterize translated SLC7A11 isoforms, we analyzed the human SLC7A11 mass spectrometry peptide data curated in the multi‐organism PeptideAtlas database [30]. Although the observed sequence coverage for the SLC7A11 amino acid sequence was 55%, the alignment of the observed peptides represents most of the exons, indicating comprehensive transcription of the entire sequence (Fig. S1D). To ascertain the SDS‐PAGE/WB‐displayed molecular mass of SLC7A11, we conducted an SDS‐PAGE/WB analysis of endogenous and exogenous SLC7A11 proteins. Consistently, both endogenous and exogenous SLC7A11 showed an apparent molecular mass of approximately 37 kDa, significantly lower than the calculated molecular mass of 55.4 kDa (Fig. 1B,C and Fig. S1C). Gel shift of SLC7A11 was further supported by electrophoresis migration data retrieved from the comprehensive online database of accurate electrophoresis migration patterns for human proteins, known as PUMBA [20], which indicated deviation of SLC7A11 during electrophoresis from the theoretically calculated molecular mass (Fig. 1D), with the highest peak of a molecular mass of around 36 kDa, which indicates the main band in conventional gel or Western detection (Fig. 1E). In contrast, the migration patterns for SLC3A2, which co‐express and interacts with SLC7A11 in the system Xc‐, shows the molecular mass to be consistent with its expected molecular size, and the control protein tubulin also exhibits an unaltered molecular mass. Overall, these results demonstrate that the observed gel shift for SLC7A11 is a reproducible phenomenon independent of alternative splicing and likely reflects a post‐translation modification or certain protein conformation state affecting its electrophoretic mobility.

### Proteolytic cleavage is not involved in the SLC7A11 protein processing

**Fig. 2:**
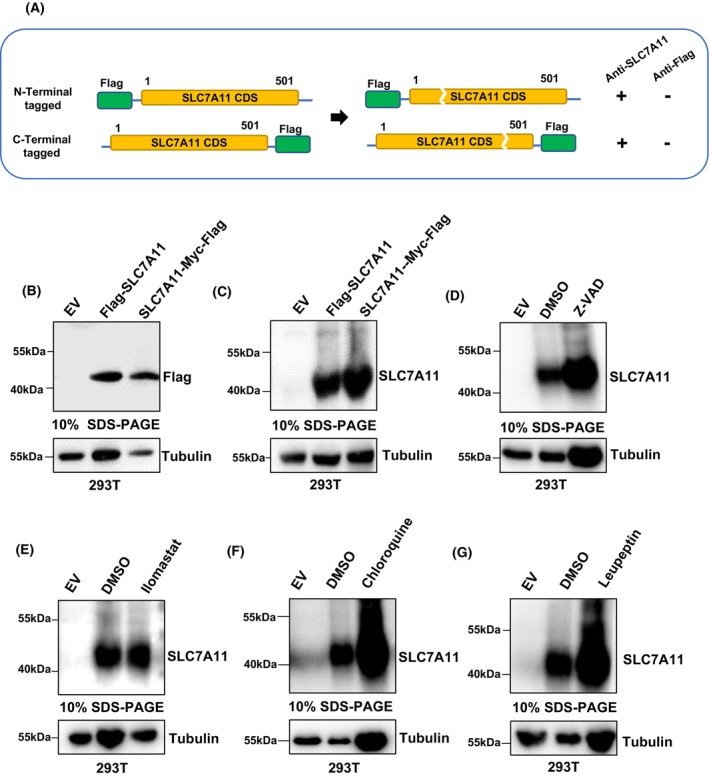
SLC7A11 protein gel shift is not associated with post‐translational proteolysis. (A) Graphical model predicting proteolytic cleavage site of SLC7A11 recombinant protein. (B, C) Detection of N‐terminal and C‐terminal flag tagged SLC7A11 indicates that SLC7A11 remained intact (not cleaved). The experiments were repeated at least two times independently, whereas representative data are shown in the text. Molecular masses presented in (B) and (C) are based on the molecular mass marker from Thermo Fisher Scientific run in parallel on the SDS gel. (D–F) SLC7A11 (SLC7A11‐Myc‐Flag) migration after protease cleavage inhibitions: protease inhibition did not revoke the gel migration deviation of SLC7A11 to actual molecular mass. Molecular masses presented in (D) to (F) are based on the molecular mass marker from Thermo Fisher Scientific run in parallel on the SDS gel. The experiments were repeated at least two times independently, whereas representative data are shown in the text. (G) Verification of post‐translational degradation of SLC7A11 (SLC7A11‐Myc‐Flag) through inhibition of autophagic proteolysis. Molecular masses presented in (G) are based on the molecular mass marker from Thermo Fisher Scientific run in parallel on the SDS gel. The experiment was repeated at least two times independently, whereas representative data are shown in the text.

PTMs affect the electrophoretic mobility of proteins in SDS‐PAGE, often resulting in aberrant molecular mass values [15, 31, 32]. To examine whether the anomalous gel mobility of SLC7A11 is associated with PTMs, we first identified the PTMs annotated for SLC7A11 in the dbPTM database that could potentially affect its gel mobility. However, these annotated PTMs typically correlate with an increased apparent molecular mass of the modified protein on SDS‐PAGE (Table S1). However, post‐translational proteolysis causes the truncated protein to migrate to a lower molecular mass position [33]. Therefore, we considered whether the low molecular mass display of SLC7A11 on SDS‐PAGE possibly results from its proteolytic cleavage, and we predicted protease cleavage sites in the SLC7A11 protein sequence using the SitePrediction webserver. The human proteases with predicted cleavage sites yielding either an N‐ or C‐terminal fragment sequence of approximately 37 kDa are presented in Table S2. Next, we aimed to determine whether the SLC7A11 proteolytic cleavage occurs by generating recombinant SLC7A11 constructs with a Flag tag at either N or C termini (Fig. 2A). SDS‐PAGE followed by WB analysis using antibodies against Flag or SLC7A11 detected both the N‐ and C‐terminal Flag‐tagged SLC7A11, displaying deviated molecular mass values of around 40 kDa (Fig. 2B,C). Then, we conducted a proteolytic cleavage inhibition assay to assess the involvement of proteolysis in the aberrant lower molecular mass display of SLC7A11 using various broad‐spectrum protease inhibitors such as leupeptin hemisulfate (50 μm), Ilomastat (50 μm) and Z‐VAD‐FMK (10 μm). To demonstrate the efficacy of the inhibitors used in this study, we pretreated cells with Z‐VAD, Ilomastat or leupeptin, followed by co‐treatment with apoptosis inducer staurosporine (5 μm). We found that Z‐VAD, Ilomastat and leupeptin could effectively inhibit the cleavage of caspase‐3 and poly (ADP‐ribose) polymerase caused by staurosporine (Fig. S2A). Despite the application of proteolytic cleavage inhibitors, the migration patterns of SLC7A11 with N‐ or C‐terminal flag were not affected at all in SDS‐PAGE (Fig. 2D–F), suggesting the unlikely event for proteolytic cleavage of SLC7A11 protein. We also treated cells with chloroquine (10 μm) and found similar results, which ruled out the effects of autophagic proteolysis for SLC7A11 (Fig. 2G). Additionally, we examined the effects of the aforementioned inhibitors on the migration of endogenous SLC7A11, and the results were consistent (Fig. S2B). These results suggest that proteolytic cleavage does not account for the anomalous lower molecular mass display of SLC7A11 on SDS‐PAGE.

### High hydrophobicity causes SLC7A11 to migrate faster in SDS‐PAGE

**Fig. 3:**
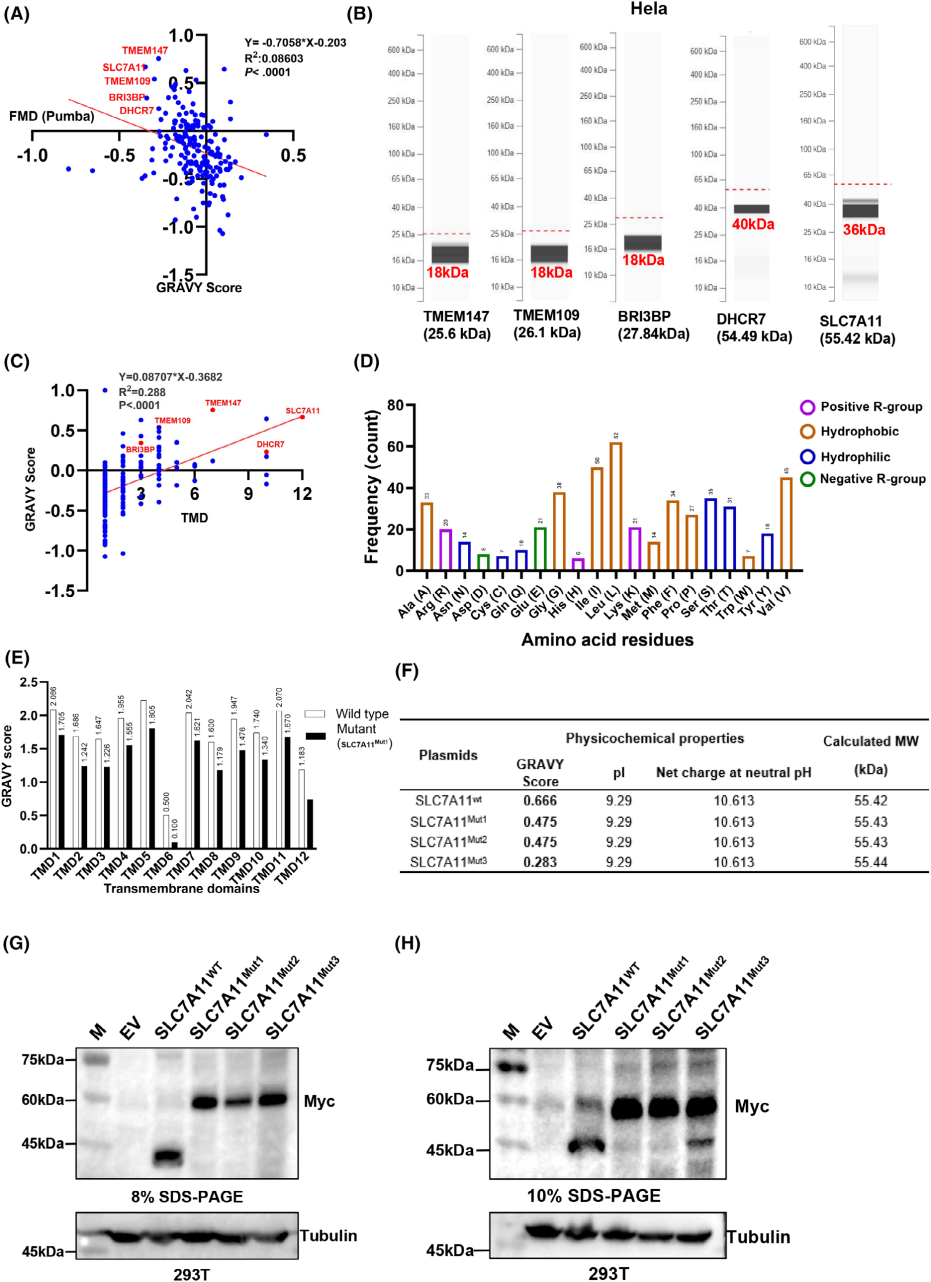
Hydrophobicity governs SLC7A11 migration in SDS‐PAGE. (A) The hydrophobicity properties of TM proteins correlate with their gel migration deviation on SDS‐PAGE. TM proteins retrieved from PUMBA database with high hydrophobicity (in red color) including SLC7A11 showed a significant correlation of hydrophobicity with faster migration (negative FMD) on SDS‐PAGE (*P* < 0.0001). FMD = [(highest peak molecular mass − theoretical molecular mass)/theoretical mass]; negative values indicate faster migration than expected; positive values indicate slower than expected. (B) High hydrophobic protein migrate faster in SDS‐PAGE to molecular mass positions that are less than their calculated molecular mass. (C) The hydrophobicity (denoted by GRAVY score) of TM proteins significantly correlate with number of TM domains. (D) Analysis of hydrophobic amino acid residues in SLC7A11. (E) Hydrophobic properties analysis for SLC7A11 TM domains shows high GRAVY score denoting high hydrophobicity; we compared SLC7A11 wild‐type TMD GRAVY score to synthesized hydrophobic mutant (SLC7A11^Mut1^, one isoleucine residue was substituted asparagine in each domain). (F) SLC7A11 hydrophobic mutants had similar physicochemical properties other than hydrophobicity. (G, H) Migration patterns of hydrophobic amino acid residue mutants and SLC7A11^WT^ (SLC7A11‐Myc‐Flag) in 8% and 10% acrylamide gels. Molecular masses presented in (G) and (H) are based on the molecular mass marker from ABclonal run in parallel on the SDS gel. The experiments were repeated at least three times independently, whereas representative data are shown in the text.

The physicochemical properties of proteins, particularly hydrophobicity, are associated with atypical gel mobility of TM protein in SDS‐PAGE [13, 34]. To investigate whether the abnormal migration of SLC7A11 in SDS‐PAGE is associated with its physicochemical properties, we first examined the correlation between hydrophobicity and migration deviation of TM proteins, including SLC7A11 using the dataset from the PUMBA database [20]. Fractionation mass deviation (FMD) refers to the migration deviation of a protein in SDS‐PAGE, and an FMD value lower than zero indicates faster migration of a protein. A high GRAVY score corresponds to the high hydrophobicity of the protein. Our analysis revealed a significant correlation between hydrophobicity and anomalous migration in SDS‐PAGE for these proteins (Fig. 3A). Virtual 1D‐SDS‐PAGE data retrieved from the PUMBA database showed a significantly deviated low molecular mass for highly hydrophobic TM proteins such as TMEM147, TM protein 109 (TMEM109), BRI3‐binding protein (BRI3BP), 7‐dehydrocholesterol reductase (DHCR7) and SLC7A11 with high GRAVY score (Fig. 3B). Furthermore, high hydrophobicity was found to correlate with increased gel mobility among TM proteins reported in various literature sources (Table S3). In addition, we observed a significant correlation between the number of TM domains and the GRAVY scores of these proteins, meaning that the protein with more TMDs is prone to have high hydrophobicity (Fig. 3C). Additionally, we noted a high number of hydrophobic amino acid residues (61.87%) in the SLC7A11 sequence, resulting in its high hydrophobic TMDs as denoted by GRAVY index scores ranging from 0.500 to 2.226 (Fig. 3C,D). To determine the influence of hydrophobicity on SLC7A11 gel mobility, we substituted non‐polar amino acid residue, isoleucine (Ile), with a strong polar asparagine (Asn) in the TMDs (SLC7A11^Mut1^, 12 mutations, one in each TMD) or extramembrane regions (SLC7A11^Mut2^, 12 mutations), whereas the SLC7A11^Mut3^ contained these mutations both in TMDs and extramembrane regions. Notably, the SLC7A11 mutants have decreased GRAVY score but similar molecular mass, pI or net charge compared with SLC7A11^wt^ (Fig. 3F). Notably, we found that SLC7A11^mut1^, SLC7A11^mut2^, and SLC7A11^mut3^ were subject to intracellular degradation because MG132 treatment could efficiently maintain a stable expression level of these SLC7A11 mutants (Fig. S2C). Then, samples with expressed SLC7A11^wt^ or SLC7A11^Mut^ harboring a Myc tag at its C‐terminal were separated on 8% or 10% SDS‐PAGE and immunoblotted with anti‐Myc antibodies. Importantly, the mutation of SLC7A11 hydrophobic residue restored its normal mobility in SDS‐PAGE because SLC7A11^mut1^, SLC7A11^mut2^ or SLC7A11^mut3^, rather than SLC7A11^wt^, migrated to the position corresponding to the calculated molecular mass of approximately 55.4 kDa, and the distance between SLC7A11^WT^ and its non‐hydrophobic mutants eventually decreased with an increasing acrylamide concentration (Fig. 3G,H and Fig. S2D). In addition, we also examined the effects of denaturation temperature, reducing condition and SDS reagent in the electrophoresis system. Change of denaturation temperatures (95, 37, 25 and 4 °C) and omission of the reducing agent in the sample buffer (non‐reducing buffer, without dithiothreitol) showed no significant impact on the SLC7A11 gel migration, although low denaturation temperature was found to increase the SLC7A11 level (Fig. S2E,F). We added varying SDS amounts (2%, 1%, 0.5%, 0.25% and 0% SDS) in laemmli sample buffer. After separating samples on 10% SDS‐PAGE, we found no remarkable influence of SDS concentration on SL7A11 gel migration (Fig. S2G). Collectively, these results demonstrated that high hydrophobicity of SLC7A11 is the key reason causing its anomalous migration in SDS‐PAGE.

### Acrylamide gel concentration determines the SLC7A11 migration in SDS‐PAGE

**Fig. 4:**
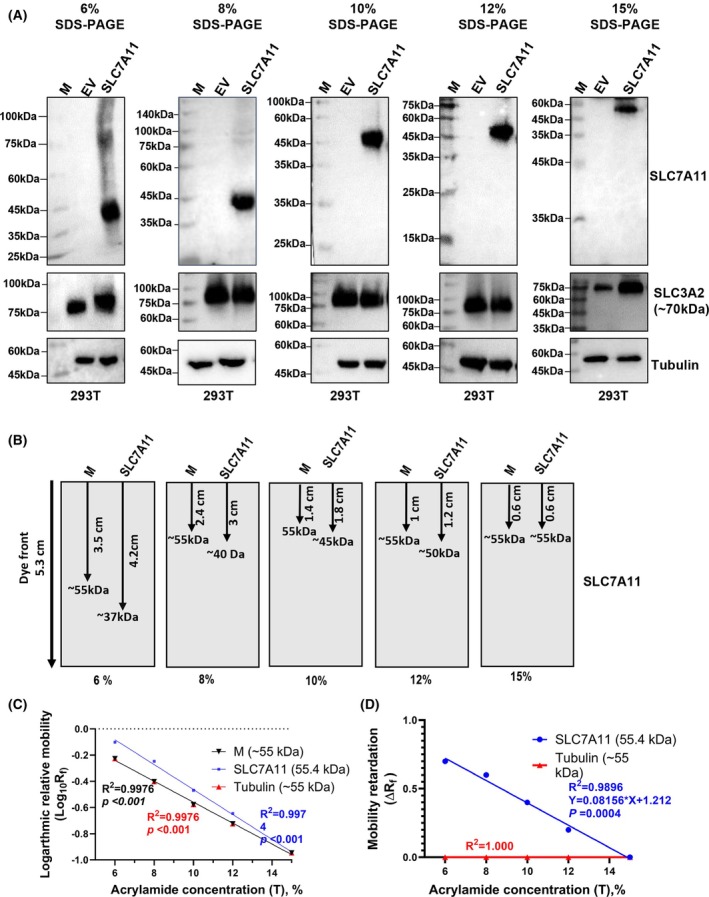
Acrylamide gel concentration determines the migration of SLC7A11 protein in SDS‐PAGE. (A) SLC7A11 (SLC7A11‐Myc‐Flag) migration pattern with ab increase in acrylamide gel concentrations. Molecular masses presented in (A) are based on the molecular mass marker from ABclonal run in parallel on the SDS gel. The experiments were repeated at least two times independently, whereas representative data are shown in the text. (B) Cartoon illustrations indicate the gel mobility of SLC7A11 on gel with varying acrylamide concentrations. (C) Ferguson plot analysis for SLC7A11, tubulin, and standard protein marker (of ~55 kDa band) electrophoretic mobility versus gel concentration. (D) SLC7A11 and tubulin mobility retardation with varying acrylamide gel concentrations.

The available spaces (pores) between the acrylamide fibers that constitute the gel matrix determine the migration speed of helical membrane proteins [21]. To investigate the SLC7A11 gel mobility with varying acrylamide gel concentrations, we systematically analyzed its migration patterns on 6%, 8%, 10%, 12% and 15% SDS‐PAGE. Notably, the migration deviation of SLC7A11 significantly decreased with an increasing acrylamide gel concentration, migrating to an approximate molecular mass position of 55 kDa on a 15% acrylamide gel, which corresponds to its theoretical molecular size of 55.4 kDa (Fig. 4A,B and Fig. S3A,B). As a control, SLC3A2 (which heterodimerizes with SLC7A11 to make system Xc^−^) and tubulin migrated to molecular mass positions equivalent to their formula calculated molecular size across all acrylamide gel concentrations (Fig. 4A). Notably, SLC3A2 is also a TM protein; however, it has a single TMD and is less hydrophobic. Therefore, this equivalent migration is attributed to their physicochemical properties, particularly low hydrophobicity (GRAVY score: −0.147 for SLC3A2 and −0.229 for tubulin). To thoroughly analyze SLC7A11 gel mobility, we generated a Ferguson plot, which is typically used to analyze the sieving properties of gel matrices. The logarithmic relative electrophoretic mobility (log_10_ *R*_f_) at different gel concentrations was plotted against gel concentrations (*T*) (Fig. 4C), indicating a significant slowed migration with an increase in acrylamide concentration, and linear regression analysis showed *R* ^2^ values ranging from 0.9974 to 0.9976 and a significant non‐zero slope of *P* < 0.0001. The SLC7A11 mobility retardation significantly (*P* = 0.004) decreased with an increasing acrylamide gel concentration, reaching zero mobility retardation on 15% SDS‐PAGE (Fig. 4D). Moreover, to exclude the possibility that the migration pattern of SLC7A11 is affected by protein markers, we employed protein markers from a different vendor and found consistent results, meaning that SLC7A11 migrated faster in low‐concentration gels, but moved equivalently with the reference markers having similar molecular mass with SLC7A11 in high‐concentration (12% or 15%) acrylamide gel (Fig. S3A,B). Overall, these findings indicate the influence of concentration for acrylamide gel and highlight its sieving property difference which may affect the migration capacity of SLC7A11.

## Discussion

The present study investigated the potential causes for the anomalous gel migration of SLC7A11, including the expression of different protein isoforms, post‐translational modifications, proteolytic cleavage and physicochemical properties such as hydrophobicity. This anomalous gel mobility was confirmed by annotated SLC7A11 migration patterns retrieved from the PUMBA database [20], which indicated the predominant migration patterns of approximately 36 kDa. Besides protein molecular mass estimation, SDS‐PAGE gel mobility often provides valuable information on protein structure complexes, protein isoforms, physicochemical properties, and PTMs [13, 15]. However, in the present study, the proteolytic cleavage is unlikely to contribute to the deviated gel mobility of SLC7A11 because the antibody against Flag could detect both N‐ or C‐terminal tagged SLC7A11 (Fig. 2A–C). Principally, the present study findings suggest the high hydrophobicity of SLC7A11 as the leading cause of its anomalous mobility in SDS‐PAGE, as evidenced by the electrophoretic gel mobility shift disappearing after the mutational decrease in hydrophobicity for SLC7A11 (Fig. 3F–H), which exclusively explains the reason for the gel shift of SLC7A11 in the field.

The characterization of membrane proteins is of significant interest as a result of their critical roles in molecule transport and drug delivery into cells. However, strong hydrophobic residues embedded in the membrane lipid bilayer present challenges for solubilization and the application of standard proteomics methods such as SDS‐PAGE, 2DE‐PAGE, liquid chromatography, mass spectrometry and X‐ray crystallography and lead to unexpected results such as gel shift [35, 36, 37]. Physicochemical properties analysis indicates a high hydrophobicity of SLC7A11, with a high GRAVY score of 0.666% and 61.87% hydrophobic amino acid residues. The substitution of SLC7A11 non‐polar, isoleucine (Ile) with polar, asparagine (Asn) amino acid residues resolved the SLC7A11 actual migration in SDS‐PAGE, migrating to a molecular mass position approximate to the formula calculated molecular mass of approximately 55.4 kDa. This slowing down of gel migration of SLC7A11 with substitution of non‐polar residues by polar residues corroborates previous findings reporting that the polar residue within a TMD alters the nature of the interaction between the TM segment and the solvating detergent, subsequently changing their electrophoretic mobility. The assessment of detergent amount binding differences in the presence versus absence of polar amino acids in the TM segment showed decreased detergent binding with a loss of approximately four detergent molecules upon inclusion of a single polar residue asparagine [38]. Furthermore, SDS‐PAGE analysis indicated a slow migration rate with the introduction of a single Asn residue into the common surface of the more hydrophobic peptide whereas the inclusion of an Asn residue in the less hydrophobic peptides increased gel migration [39, 40]. On the other hand, a previous study reported an increased gel migration rate with Ile‐to‐Asn substitution, and this increased migration was observed with substitutions located at or near the center of TM helix sequence [41], which also indicates the effect of polar amino acid position to TM protein migration, as well as interaction with detergents. SDS, an extensively studied ionic detergent, preferably interacts with the protein hydrophobic residues. Typically, the maximum SDS‐binding level among proteins is estimated at 1.4 g SDS·g^−1^ protein (one molecule/two amino acids) [42]. However, the TM protein binds two‐fold greater amounts of SDS than the globular proteins [43]. Studies have reported a correlation between the SDS‐binding capacity of highly hydrophobic proteins and an increased gel mobility for different helical membrane proteins [13]. Nevertheless, SDS binding is not the reason for the aberrant migration of SLC7A11 in SDS‐PAGE because the removal of SDS from either the sample‐loading buffer or running buffer did not change the expression pattern of SLC7A11 in acrylamide gel (Fig. S2G).

Our results revealed the fact that SLC7A11 migration in SDS‐PAGE is influenced by the acrylamide gel concentration, meaning that SCL7A11 migrated faster on low acrylamide‐concentrated gels, displaying a deviated molecular mass, whereas, on 12% or 15% SDS‐PAGE, there was a decreased mobility deviation with zero mobility retardation (Δ*R*_f_ = 0) for SLC7A11 and it displayed a molecular mass of approximately 55 kDa, equivalent to its calculated molecular mass (Fig. 4A–D). A previous study reported decreased mobility deviation for TM protein mimetic PL20 containing core sequence NH_2_‐SKSKS‐Leu_20_‐SKSKS‐NH_2_ via SDS‐PAGE with an increase in acrylamide gel concentration [21]. Moreover, the deviated molecular mass display on SDS‐PAGE for TM protein has also been linked to differences of physicochemical properties, especially high hydrophobicity and detergent binding capacity between these TM proteins and the routinely used reference proteins for monitoring molecular mass on SDS‐PAGE. Typically, estimating the molecular mass of proteins on SDS‐PAGE requires the analyte protein and the calibration reference proteins to have similar conformation in the SDS detergent treatment, as well as to be uniformly sieved through the gel matrix. Occasionally, the apparent molecular mass observed on the gel may not always correspond exactly to the actual molecular mass of the reference protein markers because of various factors such as the protein's shape and hydrophobicity, and how it interacts with the gel matrix. The calibration of SDS‐PAGE with mimetic hydrophobic standard ‘Poly‐Leu’ consisting of hydrophobic peptides “’_2_N‐Cys‐Ser‐LysSer‐Lys‐Ser‐(Leu)n‐Ser‐Lys‐Ser‐Lys‐Ser‐Cys‐NH_2_” mimicking high hydrophobicity and leucine abundance in helical TM sequence showed the reduced apparent migration deviation for helical membrane proteins to approximately 7% compared to 20% deviation from the actual value observed with commercial standards [44]. Thus, the present study also highlights the necessity of the appropriate type of protein markers for analyzing proteins with certain features including high hydrophobicity; otherwise, the unexpected migration pattern of proteins in SDS‐PAGE might mislead our research and result in tremendous costs with respect to time and money.

The change in gel mobility with varying acrylamide concentrations might be attributed to factors that affect the size and shape of the protein–SDS complex, thereby affecting its interaction with the available space in the gel matrix, including detergent–protein conformation, stoke radius, helicity and changes in the mass/charge ratio [16], which may differ from analyte proteins and reference proteins. These factors can account for the observed changes in gel migration of SLC7A11 with different acrylamide concentrations, which awaits further investigation. Based on the above results, we suggest using the varied acrylamide concentrated gel to determine the equivalent gel mobility for both SLC7A11 and a reference protein. Otherwise, the hydrophobic protein standards should be used to monitor SLC7A11 expression, although commercially available protein markers typically do not include hydrophobic proteins. In summary, we have revealed that the high hydrophobicity of SLC7A11 is the main factor associated with its atypical faster migration to low molecular mass in SDS‐PAGE. Our data suggest the important effects of physicochemical properties with respect to analyzing protein expression and provide insights into achieving accurate protein characterization by changing experimental conditions such as the acrylamide gel concentration, especially for TM proteins exhibiting atypical electrophoretic gel mobility.

## Conflicts of interest

The authors declare that they have no conflicts of interest.

### Peer review

The peer review history for this article is available at https://www.webofscience.com/api/gateway/wos/peer‐review/10.1002/2211‐5463.70019.

## Author contributions

NE, QH, YK, DZ, MG, MW, KF, SW, BF and JX collaboratively conducted the experiments. YZ, ZX and JY supervised the work. NE and QH wrote the manuscript and organized the figures. YZ, JY and YN reviewed and edited the figures and manuscript and also interpreted the data. All authors have reviewed and approved the final version of the manuscript submitted for publication.

## Supporting information


**Fig. S1.** Comprehensive analysis of SLC7A11 transcript variants and protein isoform.
**Fig. S2.** Investigation of sample preparation effects on SLC7A11 gel migration.
**Fig. S3.** Acrylamide gel concentration determines SLC7A11 migration on SDS‐PAGE.
**Table S1.** Databases annotated post‐translational modifications of SLC7A11.
**Table S2.** Proteolytic cleavage site prediction for SLC7A11.
**Table S3.** Correlation of hydrophobicity and gel shift among globular and transmembrane proteins, along with SLC7A11.

## Data Availability

The authors declare that all data that support the findings of this study are available upon reasonable request.
