# Supplementary material for: Hydrophobicity causes anomalous migration of cystine/glutamate antiporter SLC7A11 in SDS‐PAGE with low acrylamide concentration
Source: FEBS Open Bio. 2025 Mar 24;15(6):994–1008. doi: 10.1002/2211-5463.70019 (PMC12127883; doi:10.1002/2211-5463.70019)
Supplement: Supplementary file 1 — Fig. S1. Comprehensive analysis of SLC7A11 transcript variants and protein isoform. Fig. S2. Investigation of sample preparation effects on SLC7A11 gel migration. Fig. S3. Acrylamide gel concentration determines SLC7A11 migration on SDS‐PAGE. Table S1. Databases annotated post‐translational modifications of SLC7A11. Table S2. Proteolytic cleavage site prediction for SLC7A11. Table S3. Correlation of hydrophobicity and gel shift among globular and transmembrane proteins, along with SLC7A11. [file FEB4-15-994-s001.zip › feb470019-sup-0008-Supporting information.pdf]

Table S1: Databases annotated posttranslational modifications of SLC7A11

PTMs	Position
Ubiquitination	Lys^{4}, Lys^{12}, Lys^{30}, Lys^{41}, Lys^{43}, Lys^{222}, Lys^{483}, Lys^{500}
Phosphorylation	Ser^{8}, Thr^{9}, Ser^{11}, Tyr^{15}, Ser^{26}, Ser^{481}
N-linked glycosylation	Asp^{314}
S-palmitoylation	Cys^{327}

The annotated posttranslational modifications of SLC7A11 were retrieved from dbPTM database (https://awi.cuhk.edu.cn/dbPTM/index.php).

Table S2: Proteolytic cleavage site prediction for SLC7A11

Protease Merops ID	Enzymes (Homosapiens 4.2)	Cleavage at C terminal fragment				Cleavage at N terminal fragment			
		Prediction site (P4-P2',P4')	Prediction site (P4-P2',P4')	N fragment size (kDa)	C fragment size (kDa)	Prediction site (P4-P2',P4')	Prediction site (P4-P2',P4')	N fragment size (Kda)	C fragment size (kDa)
C02.001	Calpain-1	337 to 342	AVSR/LF	36.8	18.6	183 to 188	SVSW/SA	19.9	35.5
C02.002	Calpain 2	-	-	-	-	161 to 166	PELA/IK	17.6	37.8
C14.003	Caspase 3	345 to 350	ASRE/GH	37.8	17.6	159 to 164	EIPE/LA	17.4	38
C14.004	Caspase 7	345 to 350	ASRE/GH	37.8	17.6	159 to 164	EIPE/LA	17.4	38
C14.010	Caspase 9	320 to 325	PIFV/ALSC	35.2	20.2	167 to 172	LITA/VGI	18.2	37.2
CO1.060	Cathepsin B	337 to 342	AVSR/LF	36.8	18.6	-	-	-	-
A01.009	Cathepsin D	333 to 338	GGVF/AV	36.4	19	171 to 176	VGIT/VV	18.6	36.8
A01.010	Cathepsin E	339 to 344	SRLF/YV	37.1	18.3	167 to 172	LITA/VG	18.2	37.2
SO1.133	Cathepsin G	333 to 338	GGVF/AV	36.4	19	177 to 182	MVLN/SM	19.2	36.2
C01.036	Cathepsin K	350 to 355	HLPE/IL	38.3	17.1	167 to 172	PELA./IK	17.6	37.8
M10.001	MMP1	337 to 342	AVSR/LF	36.8	18.6	169 to 174	TAVG/IT	18.4	37
M10.005	MMP3	334 to 339	GVFA/VS	36.5	18.9	169 to 174	TAVG/IT	18.4	37
M10.008	MMP7	337 to 342	AVSR/LF	36.8	18.6	159 to 164	EIPE/LA	17.4	38
M10.004	MMP9	334 to 339	GVFA/VS	36.5	18.9	169 to 174	TAVG/IT	18.4	37,0
M10.009	MMP12	334 to 339	GVFA/VS	36.5	18.9	-	-	-	-
MB.001	Neprilysin	324 to 329	ALSC/FG	35.5	19.9	-	-	-	-
S08.073	Furin	344 to 351	VSRL/FYVA	37.7	17.7	-	-	-	-
S08.074	PC4	344 to 351	VASR/EGHL	36,8	17.7	-	-	-	-
S08.039	PCSK9	335 to 342	VFAV/SRLF	36.6	18.8	176 to 183	VMVL/NSMS	19.1 kda	36.3
S08.075	PC5	337 to 344	AVSR/LFYV	36.8	18.6	-	-	-	-
S08.077	PC7	337 to 344	AVSR/LFYV	36.8	18.6	-	-	-	-

The proteolytic cleavage sites of SLC7A11 were predicted using an online webserver SitePrediction (https://www.dmbr.ugent.be/prx/bioit2-public/SitePrediction/). The presented proteases enzymes are proteases with the predicted cleavage site which may release either an N or C terminal fragment with a size of approximately 37 kDa. Merops ID defines the protease identity and classification in MEROPS, the peptidase database (https://www.ebi.ac.uk/merops/index.shtml).MMP: matrix metalloproteinase; PC: proprotein convertase; PCSK: proprotein convertase subtilisin/kexin.

Table S3: Correlation of hydrophobicity and gel shift among globular and transmembrane proteins, along with SLC7A11

Protein name	UniProt accession No.	Amino acids	GRAVY index score	Calculated MW (kDa)	Displayed MW (kDa)	Membrane domains
**SLC7A11**	**Q9UPY5**	**501**	**0.666**	**55.42**	**~37[1][2]**	**12**
GAPDH	P04406	335	-0.108	36.05	~37[3]	0
Tubulin	Q71U36	451	-0.229	50.1	~55[4][1]	0
Vinculin	P18206	1134	-0.405	123.79	~124[1]	0
Actin	P60708	375	-0.2	41.73	~42[5]	0
Solute carrier family 3 member 2 (SLC3A2)	P08195	630	-0.147	67.99	~70[6]	1
GRIK4	Q16099	956	-0.047	107	~103[7]	3
NOX4	Q9NPH5	578	-0.052	66.93	~67[7]	6
ATP1A1	P05023	1023	0.011	112.8	~110[7]	10
MCT2	Q60669	478	0.363	52	~52[7]	12
Ferroportin	Q9NP59	571	0.423	62.54	~62[8]	12
GLUT1 (SLC2A1)	P11166	492	0.534	54.08	~54[9]	12
SGLT1(SLC5A1)	P13866	664	0.563	73.49	~74[10]	14
GLUT4 (SLC2A4)	P14672	509	0.559	54.78	~54[5]	12
**Protein translocase subunit SecY***	**Q5SHQ8**	**438**	**0.672**	**48.204**	**~33.5[11]**	**10**
**Cytochrome c oxidase, cbb3-type, subunit N***	**D9IA43**	**474**	**0.604**	**52.78**	**~41[11]**	**12**
**Hydantoin transport protein***	**D6R8X8**	**489**	**0.714**	**53.33**	**~36[11]**	**12**
**Arginine/agmatine antiporter***	**P60063**	**445**	**0.892**	**46.84**	**~31.8[11]**	**12**
**L-fucose-proton symporter***	**P11551**	**438**	**0.705**	**47.4**	**~32[11]**	**12**
**H(+)/Cl(-) exchange transporter ***	**P37019**	**473**	**0.63**	**50.34**	**~38[11]**	**10**
**Ubiquinol oxidase subunit 1***	**P0ABI8**	**663**	**0.658**	**74.36**	**~58[11]**	**12**

Table S3: Transmembrane proteins with high hydrophobicity exhibit anomalous gel migration on SDS-PAGE (proteins in bold). GRAVY score and MW were computed with ProtoParam, the number of transmembrane domains for human protein were retrieved from Human Transmembrane Protein database (https://htp.unitmp.org/), and the apparent molecular weight were received from literatures (indicated references). * Bacteria transmembrane proteins, Positive and Negative GRAVY index score denotes the hydrophobic and hydrophilic polypeptide respectively.

References

1. Zhang, Y., Swanda, R. V., Nie, L., Liu, X., Wang, C., Lee, H., Lei, G., Mao, C., Koppula, P., Cheng, W., Zhang, J., Xiao, Z., Zhuang, L., Fang, B., Chen, J., Qian, S. B., and Gan, B. (2021) mTORC1 couples cyst(e)ine availability with GPX4 protein synthesis and ferroptosis regulation. Nat. Commun. 10.1038/s41467-021-21841-w
2. Yan, Y., Teng, H., Hang, Q., Kondiparthi, L., Lei, G., Horbath, A., Liu, X., Mao, C., Wu, S., Zhuang, L., James You, M., Poyurovsky, M. V., Ma, L., Olszewski, K., and Gan, B. (2023) SLC7A11 expression level dictates differential responses to oxidative stress in cancer cells. Nat. Commun. 14, 1–15
3. Yang, H., Sun, W., Bi, T., Wang, Q., Wang, W., Xu, Y., Liu, Z., and Li, J. (2023) The PTBP1-NCOA4 axis promotes ferroptosis in liver cancer cells. Oncol. Rep. 49, 1–11
4. Parker, A. L., Teo, W. S., Pandzic, E., Vicente, J. J., McCarroll, J. A., Wordeman, L., and Kavallaris, M. (2018) β-Tubulin carboxy-terminal tails exhibit isotype-specific effects on microtubule dynamics in human gene-edited cells. Life Sci. Alliance. 1, 1–16
5. Hu, H., Xia, N., Lin, J., Li, D., Zhang, C., Ge, M., Tian, H., and Mei, X. (2021) Zinc Regulates Glucose Metabolism of the Spinal Cord and Neurons and Promotes Functional Recovery after Spinal Cord Injury through the AMPK Signaling Pathway. Oxid. Med. Cell. Longev. 10.1155/2021/4331625
6. Parker, J. L., Deme, J. C., Kolokouris, D., Kuteyi, G., Biggin, P. C., Lea, S. M., and Newstead, S. (2021) Molecular basis for redox control by the human cystine/glutamate antiporter system xc−. Nat. Commun. 12, 1–11
7. Tokarski, C., Fillet, M., and Rolando, C. (2011) Improved gel electrophoresis matrix for hydrophobic protein separation and identification. Anal. Biochem. 410, 98–109
8. Mesquita, G., Silva, T., Gomes, A. C., Oliveira, P. F., Alves, M. G., Fernandes, R., Almeida, A. A., Moreira, A. C., and Gomes, M. S. (2020) H-Ferritin is essential for macrophages' capacity to store or detoxify exogenously added iron. Sci. Rep. 10, 1–15
9. Oliveira, H., Roma-Rodrigues, C., Santos, A., Veigas, B., Brás, N., Faria, A., Calhau, C., de Freitas, V., Baptista, P. V., Mateus, N., Fernandes, A. R., and Fernandes, I. (2019) GLUT1 and GLUT3 involvement in anthocyanin gastric transport- Nanobased targeted approach. Sci. Rep. 9, 1–14
10. Salker, M. S., Singh, Y., Zeng, N., Chen, H., Zhang, S., Umbach, A. T., Fakhri, H., Kohlhofer, U., Quintanilla-Martinez, L., Durairaj, R. R. P., Barros, F. S. V., Vrljicak, P., Ott, S., Brucker, S. Y., Wallwiener, D., Madunić, I. V., Breljak, D., Sabolić, I., Koepsell, H., Brosens, J. J., and Lang, F. (2017) Loss of Endometrial Sodium Glucose Cotransporter SGLT1 is Detrimental to Embryo Survival and Fetal Growth in Pregnancy. Sci. Rep. 7, 1–10
11. Rath, A., and Deber, C. M. (2013) Correction factors for membrane protein molecular weight readouts on sodium dodecyl sulfate-polyacrylamide gel electrophoresis. Anal. Biochem. 434, 67–72

Fig.S1

A

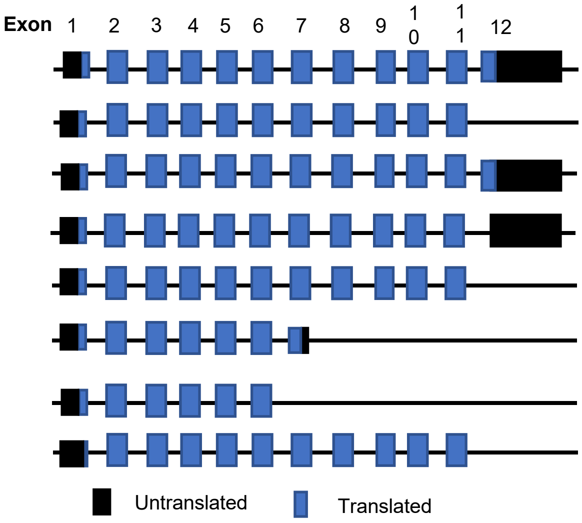

	NCBI accession number		Nucleotides	Encoded Amino acids	Theoretical pI/MW:
	NM_014331.1	NP_055146.1	1506 nt	501 aa	9.29 / 55423.03 Da
	XM_047449956.1(X1)	XP_047305912.1	1563 nt	520 aa	9.40 / 57556.42 Da
	XM_047449957.1(X2)	XP_047305913.1	1548 nt	515 aa	9.21 / 56933.72 Da
	XM_011531802.4 (X3)	XP_011530104.1	1521 nt	506 aa	9.46 / 56045.74 Da
	XM_047449958.1(X4)	XP_047305914.1	1491 nt	496 aa	9.36 / 54684.08 Da
	XM_054349497.1(X5)	XP_054205472.1	948 nt	315 aa	9.11/34788.21 Da
	XM_054349498.1 (X6)	XP_054205473.1	858 nt	285 aa	9.38 / 31141.77 Da
	XM_054349496.1 (X7)	XP_054205471.1	1618 nt	429 aa	8.91 / 48040.24 Da

B

CCDS	Current	Status	Species	Chrom.	Gene	CCDS Release	NCBI Annotation Release	Ensembl Annotation Release
3742.1		Public	*Homo sapiens*	4	SLC7A11	1	35.1	23
3742.1		Public	*Homo sapiens*	4	SLC7A11	3	36.2	41
3742.1		Public	*Homo sapiens*	4	SLC7A11	5	36.3	47
3742.1		Public	*Homo sapiens*	4	SLC7A11	6	37.1	55
3742.1		Public	*Homo sapiens*	4	SLC7A11	8	37.2	62
3742.1		Public	*Homo sapiens*	4	SLC7A11	9	37.3	64
3742.1		Public	*Homo sapiens*	4	SLC7A11	11	103	68
3742.1		Public	*Homo sapiens*	4	SLC7A11	12	104	71
3742.1		Public	*Homo sapiens*	4	SLC7A11	14	105	73
3742.1		Public	*Homo sapiens*	4	SLC7A11	15	105	74
3742.1		Public	*Homo sapiens*	4	SLC7A11	17	106	76
3742.1		Public	*Homo sapiens*	4	SLC7A11	18	107	79
3742.1		Public	*Homo sapiens*	4	SLC7A11	20	108	85
3742.1		Public	*Homo sapiens*	4	SLC7A11	22	109	92
3742.1	✔	Public	*Homo sapiens*	4	SLC7A11	24	110	108

C

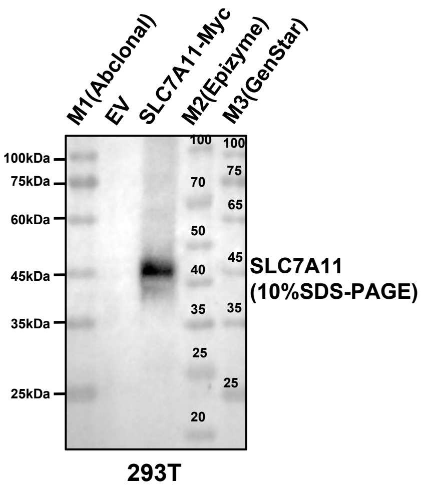

D

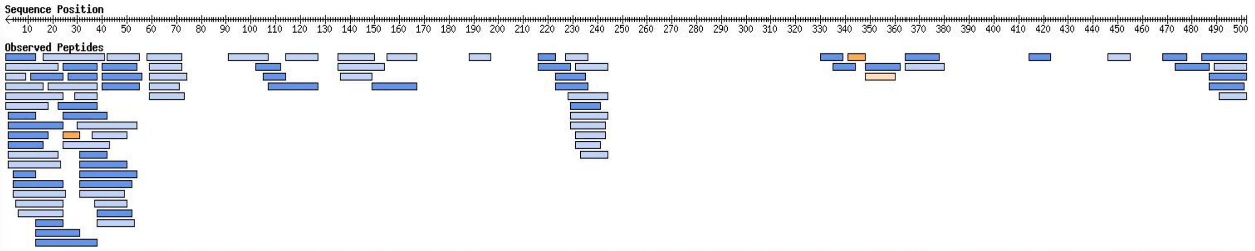

MVRKPVVSTISKGGYLQGNVNGRLPSLGNKEPPGQEKVQLKRKVTLLRGVSIIIGTIIGAGIFISPKGVLQNTGSVGMSLTIWTVCGVLSLFGALSYAEL 100
GTTIKKSGGHYTYILEVFGPLPAFVRVWVELLIIRPAATAVISLAFGRYILEPFFIQCEIPELAIKLITAVGITVVMVLNSMSVSWSARIQIFLTFCKLT 200
AILIIIVPGVMQLIKGQTQNFKDAFSGRDSSITRLPLAFYYGMYAYAGWFYLNFVTEEVENPEKTIPLAICISMAIVTIGYVLTNVAYFTTINAEELLLS 300
NAVAVTFSERLLGNFSLAVPIFVALSCFGSMNGGVFAVSRLFYVASREGHLPEILSMIHVRKHTPLPAVIVLHPLTMIMLFSGDLDSLLNFLSFARWLFI 400
GLAVAGLIYLRYKCPDMHRPFKVPLFIPALFSFTCLFMVALSLYSDPFSTGIGFVITLTGVPAYYLFIIWDKKPRWFRIMSEKITRTLQIILEVVPEEDK 500
L

Protein Coverage = **55%** (61.6% of likely observable sequence)

Figure S1. Comprehensive Analysis of SLC7A11 Transcript Variants and Protein Isoform: (A) The cDNA sequences of the predicted variants SLC7A11 from National Center for Biotechnology Information(NCBI) database were aligned with the canonical SLC7A11 Transcript (NM_014331.1). Blue boxes represent exons connected by introns (black lines), and black boxes indicate non-translated region sequences [3' and 5' untranslated region(UTR)]. Canonical and predicted transcript variants along with their encoded protein accession numbers and molecular sizes are presented. **(B)** The Consensus Coding Sequence (CCDS) database consistently reports a single coding sequence for SLC7A11 (CCDS3742.1) across all releases. **(C)** The apparent MW of SLC7A11 (SLC7A11-Myc-Flag) was determined by SDS-PAGE using different MW protein markers M1 to M3 (M1: ABclonal, M2: Epizyme, and M3 Genstar). The experiments were repeated at least two times independently, while the representative data was shown in the manuscript. (**D**) Mass Spectrometry (MS) data from the PeptideAtlas database shows low sequence coverage of SLC7A11**.** The upper bars represent observed peptides mapped onto the SLC7A11 sequence with red amino acids residues highlighting the covered sequences.

Fig.S2

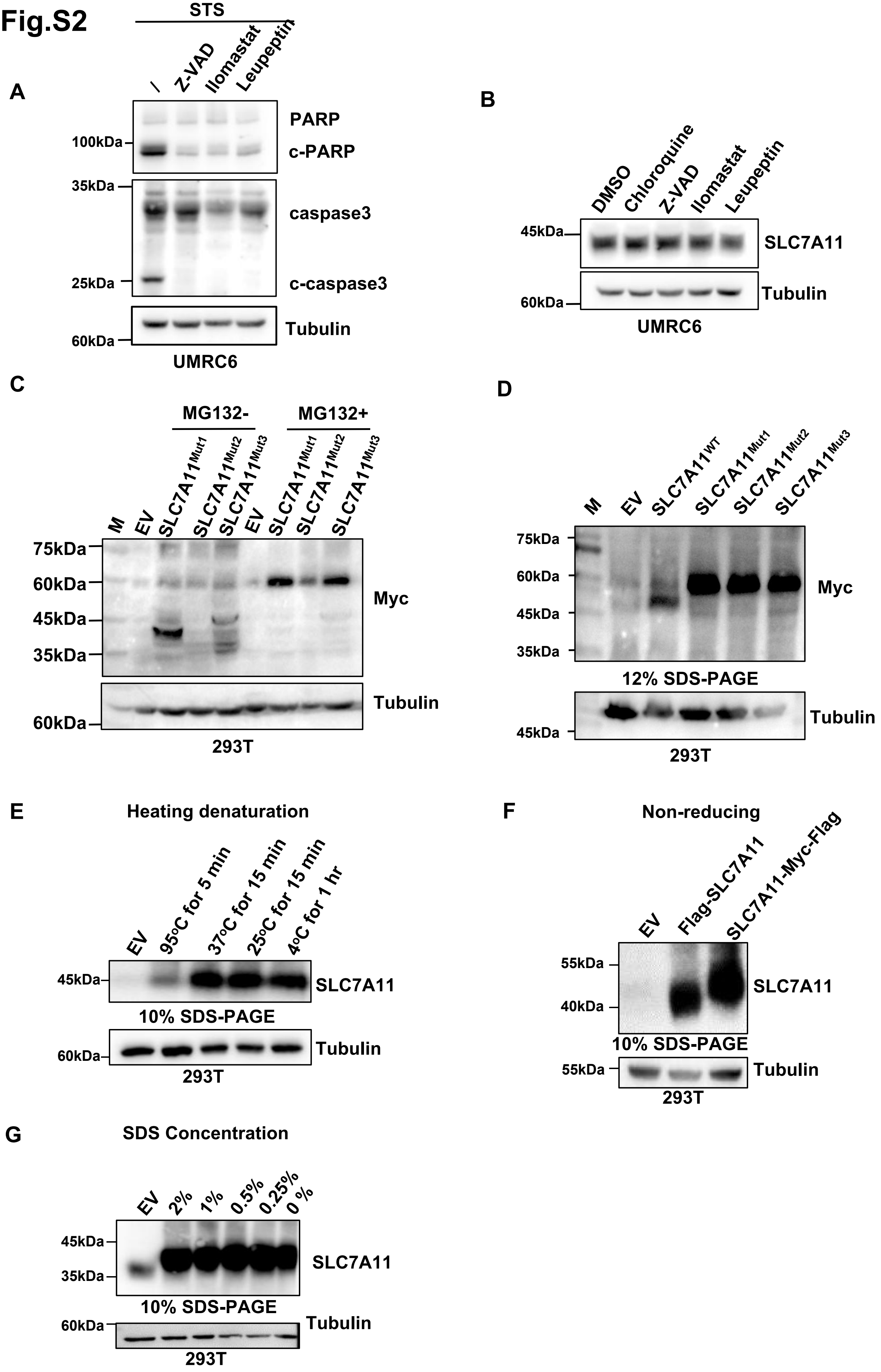

A
STS
/
Z-VAD
Ilomastat
Leupeptin
100kDa
35kDa
25kDa
60kDa
PARP
c-PARP
caspase3
c-caspase3
Tubulin
UMRC6
B
DMSO
Chloroquine
Z-VAD
Ilomastat
Leupeptin
45kDa
60kDa
SLC7A11
Tubulin
UMRC6
C
MG132-
MG132+
M
EV
SLC7A11^Mut1
SLC7A11^Mut2
SLC7A11^Mut3
EV
SLC7A11^Mut1
SLC7A11^Mut2
SLC7A11^Mut3
75kDa
60kDa
45kDa
35kDa
60kDa
Myc
Tubulin
293T
D
M
EV
SLC7A11^WT
SLC7A11^Mut1
SLC7A11^Mut2
SLC7A11^Mut3
75kDa
60kDa
45kDa
35kDa
45kDa
Myc
12% SDS-PAGE
Tubulin
293T
E
Heating denaturation
EV
95oC for 5 min
37oC for 15 min
25oC for 15 min
4oC for 1 hr
45kDa
60kDa
SLC7A11
10% SDS-PAGE
Tubulin
293T
F
Non-reducing
EV
Flag-SLC7A11
SLC7A11-Myc-Flag
55kDa
40kDa
55kDa
SLC7A11
10% SDS-PAGE
Tubulin
293T
G
SDS Concentration
EV
2%
1%
0.5%
0.25%
0 %
45kDa
35kDa
60kDa
SLC7A11
10% SDS-PAGE
Tubulin
293T

Figure S2. Investigation of sample preparation effects on SLC7A11 gel migration. (A) Detection of apoptotic markers by Western blotting after co-treatment with Proteolytic cleavage inhibitors and Staurosporine (5 μM) for 4 hours. (MWs presented in figure S2A is based on the MW marker from company ABclonal run in parallel on the SDS gel). **(B)** Proteolytic cleavage inhibitors do not affect the migration of endogenous SLC7A11. (MWs presented in figure S2B is based on the MW marker from company ABclonal run in parallel on the SDS gel). **(C)** Expression of SLC7A11 mutants in cells with or without MG132 treatment. (MWs presented in figure S2C is based on the MW marker from company ABclonal run in parallel on the SDS gel). **(D)**Migration patterns of hydrophobic amino acid residue mutants and SLC7A11WT (SLC7A11-Myc-Flag) in 12% acrylamide gels. (MWs presented in figure S2D is based on the MW marker from company ABclonal run in parallel on the SDS gel). **(E)**The gel mobility of SLC7A11(SLC7A11-Myc-Flag) was assessed under various denaturation conditions: the protein sample in Laemmli buffer was denatured at 95℃ for 5 minutes, at 37℃ for 15 minutes, at 25℃ for 1 hour and at 4℃ for 1 hour. (MWs presented in figure S2E is based on the MW marker from company ABclonal run in parallel on the SDS gel). **(F)** In non-reducing SDS-PAGE, SLC7A11 (Flag-SLC7A11, SLC7A11-Myc-Flag) showed a deviated molecular weight. (MWs presented in figure S2F is based on the MW marker from company Thermofisher run in parallel on the SDS gel). **(G)** The investigation of effects of varying SDS concentrations on SLC7A11(SLC7A11-Myc-Flag) gel migration, protein sample was denatured in Laemmli buffer contain different amount SDS. (MWs presented in figure S2G is based on the MW marker from company ABclonal run in parallel on the SDS gel). The experiments were repeated at least two times independently, while the representative data was shown in the manuscript.

Fig. S3

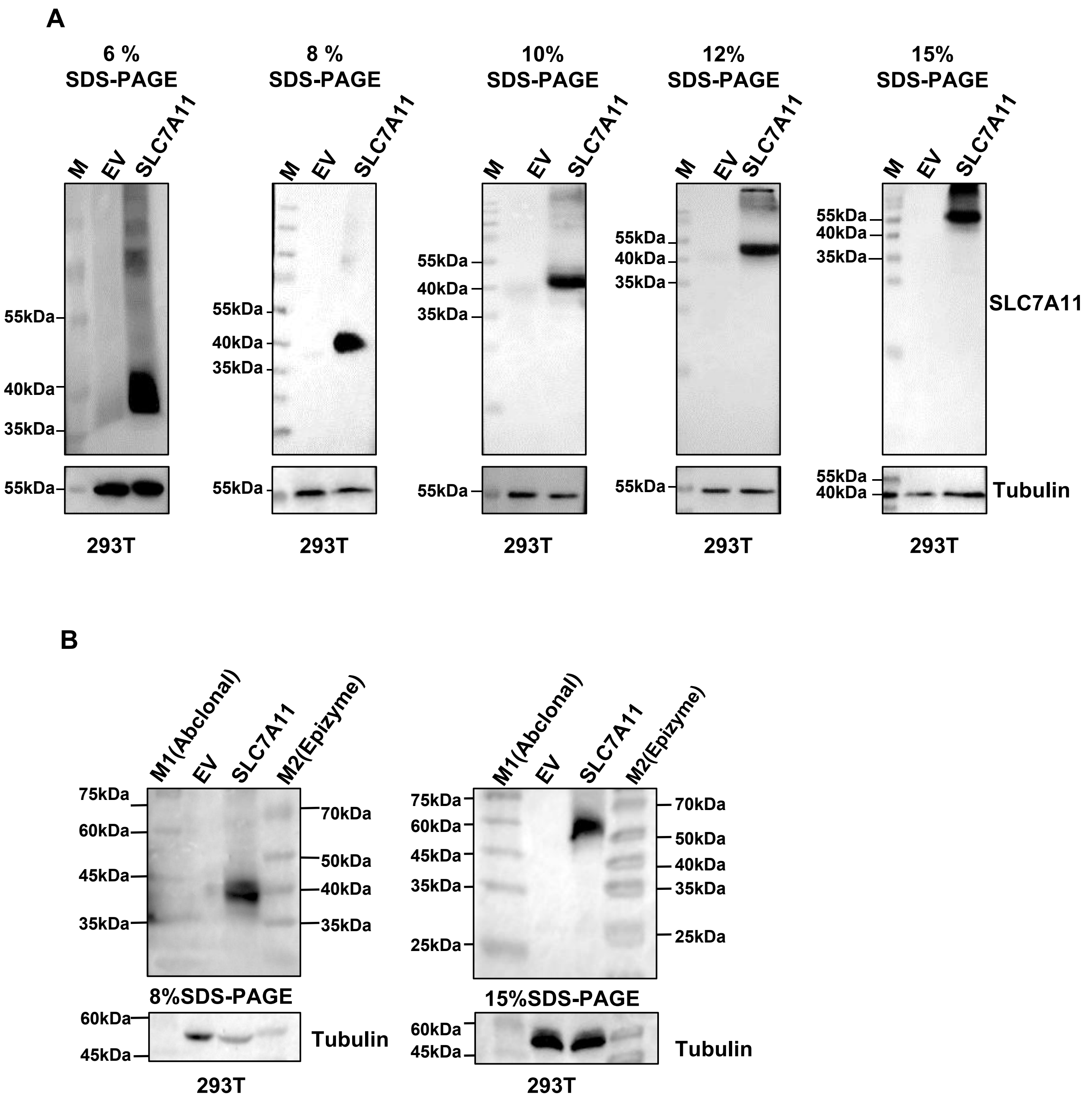

A
6 %
SDS-PAGE
8 %
SDS-PAGE
10%
SDS-PAGE
12%
SDS-PAGE
15%
SDS-PAGE
M
EV
SLC7A11
55kDa
40kDa
35kDa
SLC7A11
Tubulin
293T
B
M1(Abclonal)
EV
SLC7A11
M2(Epizyme)
75kDa
60kDa
45kDa
35kDa
25kDa
70kDa
50kDa
40kDa
35kDa
25kDa
8%SDS-PAGE
15%SDS-PAGE
60kDa
45kDa
Tubulin
293T

Figure S3. Acrylamide gel concentration determines SLC7A11 migration on SDS-PAGE. (A) SLC7A11 was separated by SDS-PAGE with indicated acrylamide concentrations, calibrated with Thermofisher MW marker Page ruler. Migration deviation decreased with increased acrylamide concentration, aligning with predicted MW of approximately 55 kDa on 15% acrylamide gel. **(B)** SLC7A11 (SLC7A11-Myc-Flag) migration positions on 8% and 15% acrylamide SDS-PAGE gels (calibrated with M1 and M2 protein markers ABclonal and Epizyme, respectively). The experiments were independently repeated at least three times, and a representative result was shown in the manuscript.
