# Supplementary figures and images for: Hydrophobicity causes anomalous migration of cystine/glutamate antiporter SLC7A11 in SDS‐PAGE with low acrylamide concentration
Source: FEBS Open Bio. 2025 Mar 24;15(6):994–1008. doi: 10.1002/2211-5463.70019 (PMC12127883; doi:10.1002/2211-5463.70019)

Fig.S2

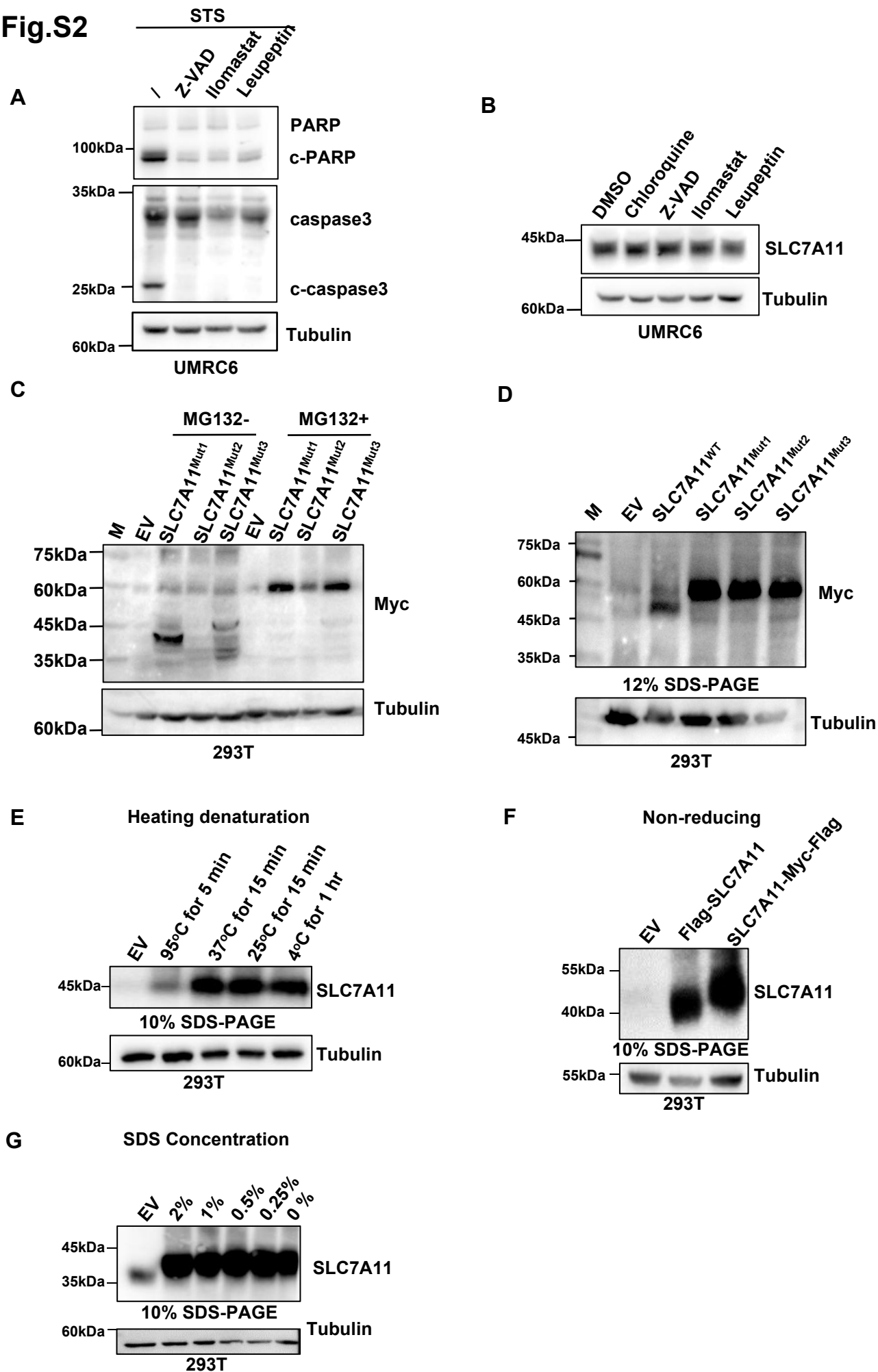

Supplement: Supplementary file 1 — Fig. S1. Comprehensive analysis of SLC7A11 transcript variants and protein isoform. Fig. S2. Investigation of sample preparation effects on SLC7A11 gel migration. Fig. S3. Acrylamide gel concentration determines SLC7A11 migration on SDS‐PAGE. Table S1. Databases annotated post‐translational modifications of SLC7A11. Table S2. Proteolytic cleavage site prediction for SLC7A11. Table S3. Correlation of hydrophobicity and gel shift among globular and transmembrane proteins, along with SLC7A11. [file FEB4-15-994-s001.zip › feb470019-sup-0003-S2 Revised.pdf]

**Fig. S3**

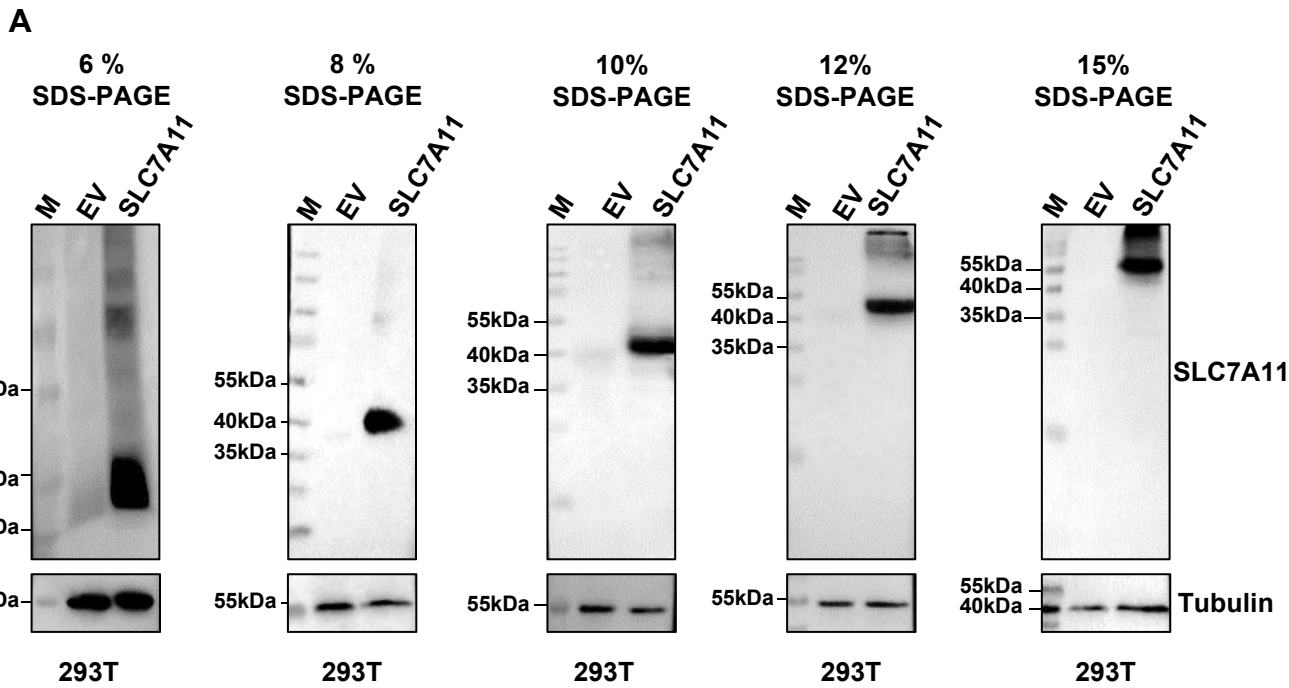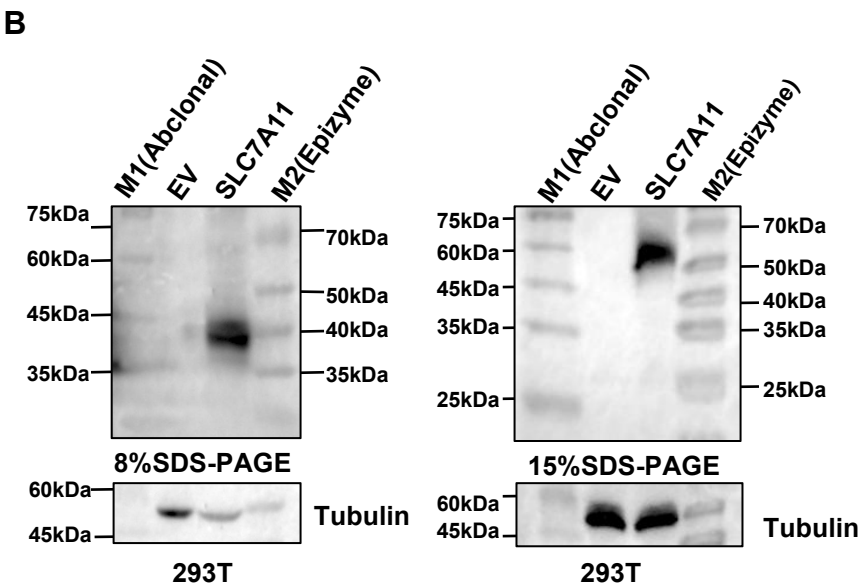

Supplement: Supplementary file 1 — Fig. S1. Comprehensive analysis of SLC7A11 transcript variants and protein isoform. Fig. S2. Investigation of sample preparation effects on SLC7A11 gel migration. Fig. S3. Acrylamide gel concentration determines SLC7A11 migration on SDS‐PAGE. Table S1. Databases annotated post‐translational modifications of SLC7A11. Table S2. Proteolytic cleavage site prediction for SLC7A11. Table S3. Correlation of hydrophobicity and gel shift among globular and transmembrane proteins, along with SLC7A11. [file FEB4-15-994-s001.zip › feb470019-sup-0004-S3 Revised.pdf]
